# The power of social talk: A longitudinal network analysis of conversations in fostering interdisciplinary collaboration

**DOI:** 10.1017/cts.2025.10124

**Published:** 2025-08-13

**Authors:** Yingshi Huang, Jinwen Luo, Vivek Shetty, Minjeong Jeon

**Affiliations:** 1 School of Education & Information Studies, University of California, Los Angeles, CA, USA; 2 School of Dentistry, University of California, Los Angeles, CA, USA

**Keywords:** Interdisciplinary, team science, longitudinal, social network, conversation

## Abstract

**Introduction:**

Interdisciplinary collaboration is essential for addressing scientific challenges, particularly in integrated fields like mobile health (mHealth), which combines computer communication and medicine to deliver healthcare services. The formation of collaborative relationships in such field is an emerging topic, with conversations among interdisciplinary scholars serving as a critical indicator of relationship development. This study aims to examine the specific effects of different conversation types (research or social oriented) on interdisciplinary collaboration and explore the impact of communication mode.

**Methods:**

We tracked conversations among interdisciplinary scholars participating in a 15-day hybrid mHealth training program, which uniquely captures both scholars’ conversation networks and the conversation quality. Three types of conversation networks were recorded (topics about current research, future research, or small talk). Using longitudinal network models, we compared the effect of different types of conversation quality on network formation and evaluated the interaction between conversation quality and communication mode (in-person or online).

**Results:**

We found that the quality of social conversations on non-research-related topics had robust effects in promoting the formation of interdisciplinary communications. In-person communication is more conducive for current and future research conversations, while online communication is valued for small talk.

**Conclusion:**

This study highlights the power of perception of personal conversation in interdisciplinary collaboration formation. The diverse effects of communication mode on different conversation networks are revealed. Our findings offer valuable insights for the event designs of interdisciplinary training program.

## Introduction

“I have read your papers, and your research is excellent!” is a common conversation starter among scholars. While praising a colleague’s work is a common icebreaker, how can scholars effectively transition from admiration to building collaborative relationships across disciplines?

The importance of this question has grown as interdisciplinary collaborations become increasingly common in scientific research [1–7], particularly in healthcare field. Interdisciplinary collaboration offers multifaceted solutions to complex healthcare problems, with each scholar contributing their own varied expertise to tackle the challenges. Integrating concepts, theories, and methods from various disciplines has been shown to foster more innovative and impactful research [8,9]. One area that greatly benefits from an interdisciplinary collaboration is mobile health (mHealth), an innovative healthcare field born from the integration of medicine and computer communication. It provides timely, location-independent solutions for medical diagnostics, disease control, and monitoring, facilitated by portable and user-friendly mobile devices [10,11]. In this study, we examine interdisciplinary collaboration in mHealth from a process-oriented perspective. Specifically, we focus on the formation process of such collaboration by tracking conversations among scholars from different disciplines. Conversational behavior is a powerful indicator for both the creation and maintenance of relationships [12–14]. It is widely acknowledged that discussing research is a highly effective way for scholars to engage each other [15,16]. However, the distinct terminologies and frameworks used in different disciplines can pose challenges in these conversations. Is it wise to focus primarily on research projects in such interdisciplinary conversations? How do different types of conversations influence the initiation and development of collaborative relationships? Hence, we aim to uncover how collaborative relationships evolve among scholars across different disciplines by evaluating the effects of conversations.

For the establishment of successful interdisciplinary team relationships, two factors are highlighted in existing research: the development of shared resources, such as shared databases and skills possessed by each team member [17,18], and the team environment, such as trust and psychological safety among members [15,19]. These two factors correspond to two key dimensions of conversation analysis, that is, informational and relational [20]. Conversations that focus on offering information and center on building relationships contribute to the team dynamics in different ways. Conversations that focus on giving or receiving information, such as discussion about research projects, can help scholars clearly know each other’s research topics and skills. If their research topics are mutually inspiring or if their skills can effectively solve problems in other fields, it incontestably facilitates the establishment of interdisciplinary collaborative relationships. Conversations that are not task- or information-oriented, such as small talk among scholars on topics unrelated to research, can enact social cohesiveness [21], nurturing the growth of team trust and comfort. Existing research on conversations mainly concentrate on the impact of self-disclosure in conversations on establishing close relationships [22,23]. Only one study [16] has attempted to analyze the relationship between STEM faculty’s job satisfaction and workplace conversations (i.e., research conversations with topics associated with work and social conversations with non-work-related topics). However, the main focus of the previous study was on conversations among faculty members with pre-existing relationships, with most interactions occurring within the same department. The role of different types of conversations in the formation of interdisciplinary collaborative relationships remains unclear. From a broader perspective, the affective primacy theory offers valuable insights. This theory emphasizes the importance of interpersonal affect in forming work-related connections [24,25]. Specifically, in the context of organizational collaboration, employees are more likely to collaborate with individuals they find pleasant to interact with rather than those they perceive as more competent. However, it remains unclear whether this conclusion can be generalized to the early stages of relationship building and the context of research collaboration. Additionally, it is uncertain how scholars decide to engage in further conversations when they feel pleasant in both task- and social-oriented interactions. That is, the quality of which types of interactions is valued more? In the context of interdisciplinary research, affective primacy theory suggests that scholars who engage in social-oriented interactions, such as small talk or informal discussions, may build greater trust and a stronger willingness to collaborate across disciplinary boundaries. For example, those who reported participating in casual, non-work-related conversations may be more likely to express interest in future collaboration, as such conversations helped foster interpersonal connection and mutual respect. Hence, challenging the traditional assumption that research-oriented conversations are preferred for establishing collaboration intentions, this study extends and validates the theory by examining it under a novel context, that is, examining the role of social-oriented conversations in the early stages of interdisciplinary collaboration.

Breaking down boundaries is a key characteristic of interdisciplinary team science, including both disciplinary boundaries and physical boundaries. Scholars from different regions are connected via online software. Hence, one central question in studying interdisciplinary conversation is how the online communication mode affects interdisciplinary team interactions compared to face-to-face interactions. Previous studies on conversations in the online mode have shown both positive and negative effects [26,27]. Through analyzing the impact of using video conferencing tools (e.g., Zoom) for social conversations, researchers found that participants found it easier to identify previously established social connections during online interactions [26]. However, difficulties in performing turn-taking behaviors and monitoring paralinguistic cues are also reported [28], implying potential effects of communication modes on the conversation quality. Although existing research has provided valuable insights into specific behaviors happening in the virtual interactions, we still lack understanding of how different communication modes affect different types of conversations. More importantly, would different modes moderate the impact of conversation types on the formation as well as maintenance of interdisciplinary collaborative relationships?

To fully decode the evolution of interdisciplinary collaborative relationships, we collected conversation data of interdisciplinary teams and employed the longitudinal network modeling approach. Social network analysis treats relational data in a view of nodes (e.g., conversation senders and receivers) and edges (e.g., presence of conversations), offering a graphical representation for the relationship among nodes and enabling detailed inspections of the dynamic relationship forming process [29]. Leveraging the longitudinal network model, we aim at (1) analyzing the development of different types of conversations (i.e., current research-oriented, future research-oriented, and social-oriented conversations) in interdisciplinary collaborations and identifying factors influencing the growth of interdisciplinary communication networks; (2) examining the impact of communication modes (i.e., online and in-person) on the development of these conversation networks and exploring the potential moderation effects. We used a data set from a mHealth training institute. The dataset offers a distinctive contribution by combining each scholar’s conversation network with scholar’s evaluations of the usefulness of the interaction, collected during the hybrid-format training program. This dataset provides an opportunity to analyze the dynamics of interdisciplinary collaboration in mobile health across both virtual and in-person settings. Additional details of the data source are provided in Section 2.1. Specifically, this study focuses on two research questions: (1) How do different types of conversations in interdisciplinary collaboration evolve over time, and more importantly, which type is most helpful for fostering future conversations? (2) How does the mode of communication influence the development of conversation networks in interdisciplinary collaboration?

Conversation is a crucial index for investigating human connections, especially in the early stages of relationship formation, where no collaborative outcomes are yet available. Whether scholars are willing to continue discussions about future research projects effectively reflects their intent to collaborate. Investigating the role of different types of conversations in interdisciplinary collaborations can reveal how the flow of various forms of information impacts team formation, functioning, and performance. Moreover, in interdisciplinary collaboration, virtual communication mode is essential for distributed teams. Understanding the impact of different communication modes on team building can help foster global team success and effectiveness. Although this study is situated within the field of mHealth, drawing on data from an mHealth training institute, its research implications may inform the session design of interdisciplinary training programs more broadly. The findings from the current study can therefore be valuable for fostering interdisciplinary collaboration in similar initiatives across other fields.

## Method

### Participants and procedures

This study leverages team process data gathered by the mHealth Training Institute (mHTI). Since 2016, the mHealth program has been linking scholars from various disciplines and universities to encourage interdisciplinary collaboration and stimulate innovation in mobile health [30]. The team process data documents daily interactions among scholars during the training, detailing who engaged with whom and the nature of these conversations. In 2023, for the first time, mHTI collected data on scholars’ perceptions of the usefulness of these conversations, offering a distinctive dataset for analysis. The 2023 training, conducted in a hybrid format (virtual and in-person), took place over 15 days from April to November. To maintain confidentiality, the data has been anonymized, ensuring that the identities of the scholars remain undisclosed while preserving the authenticity of their interactions.

**Figure 1.**
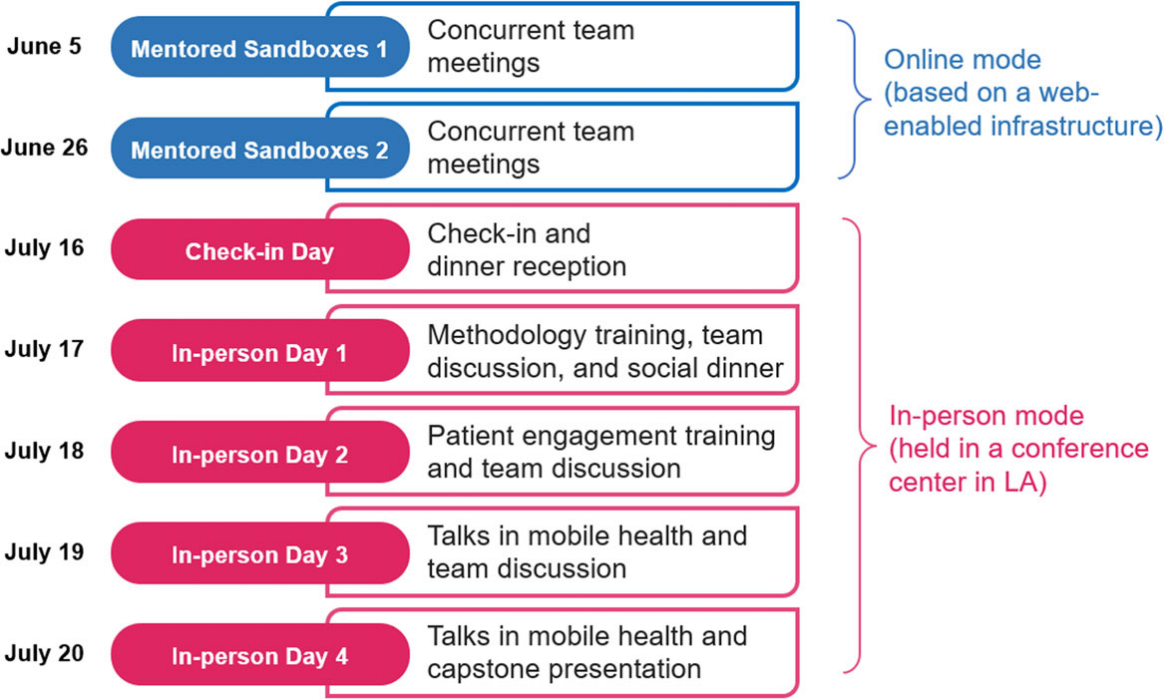
Timeline and schedule for the data collection days. LA = Los Angeles.

The 2023 program admitted 30 scholars, who attended lectures and webinars on various mHealth topics. The scholars were divided into six teams to complete a capstone project under the guidance of mentors. The teams were intentionally formed with scholars from diverse disciplines. Table 1 provides demographic information on the scholars, who come from fields such as psychology, medicine, and computer science. Notably, as shown in Table 1, in terms of career stage, 67% of the scholars were in the middle career stage (assistant or associate professor or other mid-career stage), and in terms of institution, 73% affiliated with universities. Due to the concentration of these variables, they were excluded from further analysis. Scholars were asked to report their interactions with other fellows and specify the types of conversations at seven time points (Figure 1). The first two time points were during the virtual period, which included sandbox meetings mentored by program faculty, while the latter five were during in-person events. Spread over 4 days, the in-person events included seminars, team discussions, report-backs, and final presentations of the capstone projects. At the first three time points, responses from three scholars were missing and were imputed as 0, indicating no conversations with others.

**Table 1.** Demographic characteristics for scholars

Variables		*N* (%)
Career stage	Postdoc or other early-career	6 (20%)
	Assistant or associate professor or other mid-career	20 (67%)
	Full professor or other late-career	1 (3%)
	Other	1 (3%)
	Missing	2 (7%)
Institution	Research center	5 (17%)
	University	22 (73%)
	Other	1 (3%)
	Missing	2 (7%)
Region	Northeast	6 (20%)
	Southeast	10 (33%)
	Southwest	3 (10%)
	Midwest	2 (7%)
	West	4 (13%)
	International (not from the U.S.)	3 (10%)
	Missing	2 (7%)
Race	Black/African American	3 (10%)
	White/Caucasian	12 (40%)
	Hispanic/Latina/Latino	4 (13%)
	Asian	8 (27%)
	Prefer not to state	1 (3%)
	Missing	2 (7%)
Gender	Male	9 (30%)
	Female	18 (60%)
	Prefer not to state	1 (3%)
	Missing	2 (7%)
Age group	29 or under	1 (3%)
	30 to 39	17 (57%)
	40 to 49	10 (33%)
	Missing	2 (7%)
Discipline	Psychology	13 (43%)
	Medicine / Nursing	5 (17%)
	CS / Engineering / Data Science	6 (20%)
	Public Health / Others	4 (13%)
	Missing	2 (7%)

Three types of conversation were considered: Project and Training (PT), Career, Collaboration, and Research (CCR), and Small Talk (ST). PT includes conversations about capstone projects or mHealth training, representing current research-oriented conversations. Note that the current research mainly refers to the capstone research project required by the mHealth training program; CCR contains conversations about career life and future collaborations or research beyond mHealth projects, representing future research-oriented conversations; ST refers to informal conversations unrelated to the mHealth program and research, representing social-oriented conversations. The classification of the three types of conversation aligns with research question 1. By distinguishing between small talk and research-oriented conversations, we can better differentiate the effects of informational and relational connections. The further separation between current capstone research conversations (PT) and future research-oriented conversations (CCR) allows for a more nuanced analysis of research-related conversations. This distinction explicitly captures the impact of different types of conversations on the intention and likelihood of future collaboration, facilitating a more direct assessment of how each conversation type influences the willingness to collaborate moving forward. After indicating the conversation type, scholars evaluated the degree of helpfulness of the conversation they had (1 = a little bit helpful and 4 = extremely helpful) as indicators of perceived conversation quality. Note that helpfulness is evaluated only when a conversation occurs, while not all scholars engage in conversations at each time point, and the conversations may not encompass all three types.

### Conversation networks

The conversations among scholars can be captured by a series of networks in which a network node represents a scholar and the connection, or the edge, between two nodes indicates a conversation. The conversation networks can be denoted by adjacent matrices *W*_*i*, *j*_^(*t*)^ ∈ {0, 1}^30 × 30^, where *t* = 1, 2, 3, …, 7 denotes the conversation network at time point *t*, and *i* for the conversation initiator and the *j* for the conversation receiver. At a given time point *T*, an entry *W*_*i*, *j*_^(*t*=*T*)^ = 1 indicates the scholar *i* reported that they had a conversation with *j*, and *W*_*i*, *j*_^(*t*=*T*)^ = 0 indicates that no conversation from *i* to *j* has been reported at time point *T*. Note that mHTI collected the conversation process in a way to differentiate the direction of the conversation. Scholar *i* initiating a conversation with scholar *j* does not imply that scholar *j* will also initiate a conversation with scholar *i*, signaling the features of directed networks. We further add a superscript *c* to *W*_*i*, *j*_^(*ct*)^, *c* = *PT*, *CCR*, *ST* to account for the networks of three conversation types. No attrition of participants occurred throughout the 2023 mHTI, so the size of *W* kept the same for all time points.

The conversation networks for three conversation types at seven time points are shown in Figures S1 to S3 in the supplementary material. It can be seen that during the online period (before check-in on July 16th), all three types of conversations are mostly confined within the team. Over time, the networks for all three types of conversations became more densely connected. Moreover, ST conversation networks developed more slowly in the early stages. Compared to PT and CCT networks, some teams (e.g., Team 3) had no ST interactions during the first two time points. Table 2 displays the network statistics for all three networks. The total degree is the averaged total number of adjacent edges of each node, representing the averaged popularity of nodes and the density of the network. The closeness measures the average number of steps required for each node to reach other nodes, reflecting the internal connectivity of the network and how easily a scholar can talk to other scholars; The reciprocity is the proportion of mutual conversations among scholars [31]. As in Table 2, for all conversation types, networks in the in-person mode are denser, with more connections and mutual interactions among scholars than in the online mode. Additionally, the mode effect impacts the ST network the most. Compared to PT and CCR networks, the ST network exhibits a more substantial increase in degree, closeness, and reciprocity in the in-person mode.

**Table 2.** The network statistics for project and training (PT), career, collaboration, and research (CCR), and small talk (ST) conversation networks

	PT				CCR				ST		
	total degree	closeness	reciprocity		total degree	closeness	reciprocity		total degree	closeness	reciprocity
SB - June 5	5.133	0.203	0.553		3.000	0.190	0.227		1.933	0.201	0.143
SB - June 26	4.000	0.259	0.667		2.400	0.331	0.389		2.600	0.263	0.410
Check-in July 16	4.533	0.117	0.328		4.267	0.122	0.222		7.333	0.013	0.367
Day 1 July 17	10.067	0.014	0.623		9.467	0.015	0.507		12.267	0.017	0.467
Day 2 July 18	8.600	0.048	0.775		7.733	0.036	0.603		8.867	0.014	0.571
Day 3 July 19	8.600	0.012	0.744		7.400	0.014	0.577		10.133	0.014	0.632
Day 4 July 20	8.600	0.074	0.672		7.933	0.114	0.605		10.333	0.052	0.545

### Statistical analyses

To model dynamic network structures, the Temporal Exponential Random Graph Model (TERGM) was employed [32]. Compared to traditional regression methods, TERGM directly handles network structure data and accounts for its temporal nature. It captures dependencies between the current and past states of the network, enabling us to model how early interactions influence the likelihood of future ones. Additionally, when compared to other longitudinal network models like the Stochastic Actor-Oriented Model (SAOM) [33], which primarily focuses on how node-level variables impact network structure, TERGM provides insights into the changes within the network itself, including how ties are formed. These features make TERGM particularly well-suited for studying evolving conversation patterns, as it effectively models how relationships (e.g., conversations) between individuals change over time. TERGM is a longitudinal extension of the Exponential Random Graph Model (ERGM) family. The ultimate goal of the ERGM family is to explain the probability of a connection between two nodes being present, that is, what factors contribute to the formation of an edge [34]. The potential influencing factors can be classified into three categories: individual factors, dyadic factors, and structural factors [31]. Individual factors are node attributes (i.e., node covariates), such as the gender or race of scholars; Dyadic factors describe the characteristics of the relationships between two nodes (i.e., edge covariates), such as the perceived helpfulness of the conversation between two scholars; Structural factors contain features of the entire network system, such as the number of mutual conversation in the network. To examine the effects of these factors, ERGM uses a logistic regression-like approach, treating the presence of a connection as a binary outcome variable. The key difference between logistic regression and ERGM is the dependence among outcome variables. In logistic regression, it is assumed that outcome variables are independent of each other. However, in networks, the connection between two nodes is influenced by other nodes. For example, if node *i* is connected to node *j* and node *k*, the probability of a connection between node *j* and node *k* might be high. Hence, the estimation of standard errors for regression coefficients is adjusted accordingly. ERGM focuses on the network structure at a single time point, and TERGM realizes the extension of multiple time points by incorporating the time dependence of longitudinally observed networks into the model [32]. Specifically, network statistics of networks observed at previous time points are incorporated into the determination of current network structures. The TERGM model is formulated as:

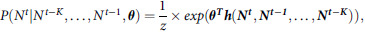




where *K* represents *K* time points previous to time *t*; ***θ*** are network parameters; *h*(⋅) are network statistics (i.e., three types of influencing factors); and *z* is the normalization term. TERGM makes no restrictions on the time intervals between observed networks. Hence, it is acceptable to build models with all conversation networks across seven time points.

Aiming to examine the impact of conversation types and training modes, we built two models for each type of conversation networks. Model 1 focuses on the effect of perceived helpfulness on the formation of future communications. Specifically, how does lagged conversation helpfulness influence the probability of forming different types of conversations at the next time point, and which type of helpfulness is the most effective in the formation of collaborative relationships? In this model, the observed conversation networks from waves 2 to 7 are used as outcome variables. Three parts of the predicted variables are considered. First, we include endogenous components of networks to control the natural tendency of networks to form different types of connections, preventing the overestimation of main effects. Four structural factors are considered: edges, the total number of connections; mutual, the total number of mutual connections; cyclicalities, the number of cyclical connections; and transitivities, the number of transitive connections [34]. Second, demographic variables are included as individual factors to investigate how scholars’ demographic profiles influence the formation of different types of conversations. We expect that the team variable will yield the strongest effect while the discipline variable will be the weakest. Next, the main predicted variables are three types of first-order lagged perceived helpfulness (waves 1 to 6), entering the model as dyadic factors. Namely, the perceived helpfulness at wave 1 is used to predict the conversation formation at wave 2, and so on. Note that perceiving one type of conversation as helpful does not necessarily imply that another type is also perceived as helpful. Therefore, the three types of helpfulness are assumed to be independent of each other, avoiding multicollinearity issues. Model 2 primarily examines the moderation effect of the communication mode, i.e., whether communication modes moderate the impact of perceived helpfulness on the formation of conversations. To answer this question, building on Model 1, Model 2 incorporates three interactions between the communication mode and helpfulness. The online mode is set as the reference and coded as 0, and the in-person mode is coded as 1. The interaction terms are computed as the product of the helpfulness matrix and communication mode, that is, equaling the helpfulness edge covariate matrix with the first two time points as 0.

We use the *btergm* R package [35] for TERGM analysis. The Markov chain Monte Carlo (MCMC) maximum likelihood estimation is implemented which demonstrates superior statistical properties with unbiased parameter estimates [35]. To assess the model fit, multivariate statistics computed from 100 simulated model-based networks are compared with those from observed networks. For directed conversation networks, distributions of six statistics are calculated: edge-wise shared partners, dyad-wise shared partners, shortest path, in-degree, out-degree, and triad census. The alignment between simulated and observed network statistics indicates desired model fit.

## Results

All models converged with well-mixed MCMC chains for all parameters, and the proposed models were well-fitted as simulated network statistics closely aligned with observed statistics (see Figures S1–S12 in the supplementary materials). The estimated parameters for two models of each conversation type are displayed in Table 3, which can be interpreted as log-odds of creating a conversation. In Model 1, we find that, as expected, all four structural variables and the team variable have significant effect on the conversation formation for all three conversation types, while the discipline effect is not significant. This indicates that the mHealth program effectively promoted all types of interdisciplinary communications among scholars. For the PT network, the lagged perceived helpfulness of PT and ST conversations can facilitate the probability of creating PT communication at the next time point by 0.545 and 0.575, respectively. If a scholar had conversations about projects and mHealth training or had unrelated casual talks with other fellows, and found these conversations helpful, the scholar is likely to discuss with these fellows about the projects and training at the next time point. Additionally, the homophily of region and race positively predicts the tie formation, meaning that scholars are more likely to talk with fellows from the same region or race about capstone projects and training. For the CCR network, both CCR and ST helpfulness are significant, that is, conversations about career life and future collaboration as well as casual topics at the previous time points can promote the probability of CCR discussion at the next time point by 0.583 and 0.546, respectively. Similarly, the region homophily can increase the probability of initiating CCR conversations. For the ST network, among all types of perceived helpfulness, only lagged ST helpfulness has a significantly positive effect on the formation of conversations (increases the probability by 0.619). Moreover, the homophily of region, race, and age shows significant effects, suggesting that scholars tend to have informal conversations with fellows who are from the same region, share the same race, and are of similar age. The strong team effect underscores the effectiveness of the mHealth program in fostering interdisciplinary communication within assigned teams.

**Table 3.** Model parameter estimates and standard errors (SE) for project and training (PT), career, collaboration, and research (CCR), and small talk (ST) conversation networks

Model 1	PT		CCR		ST	
Effect	estimates	SE	estimates	SE	estimates	SE
edges	−4.659***	0.159	−4.251***	0.128	−3.827***	0.120
mutual	2.100***	0.275	1.265***	0.243	1.208***	0.186
cyclicalties	−0.956***	0.131	−0.389***	0.098	−0.321***	0.072
transitiveties	1.432***	0.127	1.278***	0.107	1.020***	0.094
team	2.627***	0.207	1.689***	0.148	1.657***	0.136
region	0.504**	0.157	0.439**	0.137	0.261*	0.116
race	0.442**	0.139	0.218	0.124	0.250*	0.107
gender	0.098	0.119	−0.029	0.110	−0.011	0.090
age	0.109	0.129	0.098	0.116	0.206*	0.098
discipline	−0.104	0.159	0.107	0.137	0.210	0.114
PT helpfulness	0.180***	0.054	−0.070	0.049	0.024	0.048
CCR helpfulness	−0.006	0.058	0.333***	0.049	−0.047	0.050
ST helpfulness	0.301***	0.057	0.186***	0.047	0.486***	0.046
**Model 2**						
**Effect**	**estimates**	**SE**	**estimates**	**SE**	**estimates**	**SE**
edges	−4.683***	0.162	−4.246***	0.130	−3.830***	0.121
mutual	1.945***	0.285	1.234***	0.246	1.184***	0.186
cyclicalties	−0.896***	0.132	−0.408***	0.098	−0.323***	0.073
transitiveties	1.450***	0.126	1.277***	0.106	1.023***	0.098
team	2.682***	0.209	1.793***	0.150	1.705***	0.136
region	0.516**	0.159	0.468	0.133	0.278*	0.115
race	0.476***	0.139	0.247	0.126	0.262*	0.104
gender	0.086	0.120	−0.044	0.109	−0.015	0.094
age	0.133	0.135	0.109	0.121	0.225*	0.099
discipline	−0.073	0.162	0.134	0.142	0.226*	0.114
PT helpfulness	−0.019	0.075	−0.155	0.086	−0.111	0.077
CCR helpfulness	0.010	0.126	0.037	0.149	−0.066	0.145
ST helpfulness	0.329*	0.141	0.563***	0.158	0.617***	0.158
PThelpfulness *mode	0.375***	0.101	0.149	0.095	0.213*	0.088
CCR helpfulness *mode	−0.046	0.144	0.345*	0.162	0.006	0.157
ST helpfulness *mode	−0.134	0.155	−0.477**	0.169	−0.174	0.167

*Note*. *** indicates *p* < 0.001, ** indicates *p* < 0.01, * indicates *p* < .05. The significant estimates are highlighted. (online mode = 0, in-person mode = 1).

In Model 2 with interaction terms, similar to Model 1, four structural variables and the team homophily have significant effects on the formation of all types of conversations. For the PT network, after considering the mode effect, the main effect of PT helpfulness is no longer significant, while the main effect of ST helpfulness remains significantly positive. Furthermore, only the PT interaction effect reaches the significance level, indicating that compared to the online mode, in-person PT interactions are more likely to promote future conversations about projects and training. For the CCR network, the ST helpfulness main effect survives, and the CCR helpfulness main effect is insignificant. In terms of interaction effects, both interaction between CCR helpfulness and training modes and interaction between ST helpfulness and modes are significant, but in different directions. In detail, a higher perceived helpfulness of previous in-person CCR conversations can bring greater probability of having CCR conversations in the future than the online CCR helpfulness. But concerning the effect of ST, the helpfulness of online conversations can more effectively promote future CCR conversations than the helpfulness of in-person interactions. For the ST network, the main effect of lagged ST helpfulness remains significant after including interactions. Further, the interaction between PT helpfulness and training modes is significant. Namely, the lagged perceived helpfulness of in-person PT conversations can facilitate future ST conversations. Notably, after incorporating interaction effects, the discipline homophily shows a significant effect on the formation of ST conversations. This means that scholars tend to engage in informal conversations with fellows from the same discipline.

Additionally, note that the online mode includes only two time points, while the in-person mode includes five. To avoid the in-person mode dominating the analysis and to evaluate the robustness of the results, we re-estimated Model 2 using balanced-wave subsamples, specifically, two time points from the in-person mode. The estimates are presented in Table S1 of the supplementary material. The results remain robust, with the main conclusions unchanged: ST helpfulness remains a significant predictor across all three conversation types, and the interaction between ST helpfulness and communication mode is significant for CCR. Moreover, given the small number of observations in certain categories of the demographic variables, we re-estimated Models 1 and 2 by including a separate parameter for each subgroup within each demographic variable. In other words, we allowed for differential homophily, enabling each group to have its own within-group tie formation tendency. The results are presented in Tables S2 and S3. Again, the effect of small talk helpfulness remains robust in both models, as does its interaction with communication mode in predicting the CCT network. Second, across all three conversation types, participants from the South and international scholars tend to interact more with others from the same region. In terms of race, Asian scholars show a strong tendency to engage with those of the same racial background. Consistent with previous results, homophily based on age is only significant within the small talk network.

## Discussion

Interdisciplinary team collaborations have demonstrated great potential in expediting the advancement of complex scientific research, such as the emerging field of mHealth, by fostering innovative solutions and integrating diverse perspectives [8,9]. To facilitate the formation of interdisciplinary collaborations, it is crucial to examine the conversational behaviors of scholars from diverse disciplines, as these interactions hold vital information about the development of collaborative relationships. In this study, we implemented the longitudinal social network method to model three types of conversation networks among scholars from different disciplines, evaluating the effects of conversation quality and communication modes on the network formation. Intriguingly, the high perceived value of non-research-oriented social conversations consistently demonstrates positive effects in fostering various types of interactions. In contrast, the quality of research-oriented discussions only seems to encourage specific forms of dialogue. In terms of the communication mode effect, in-person conversation quality of research-oriented interactions (both conversations about current mHealth projects and future collaboration projects) is considered more valuable than online research conversations, while online conversation quality of social interaction is more effective than in-person social talks quality in the conversation network of career life and future collaborations. However, it is important to note that the current study primarily focuses on the formation of early-stage communication networks. It would be meaningful for future research to investigate how these relationships evolve into long-term collaborations and enduring partnerships.

### Conversation type effects on interdisciplinary collaborations

In interdisciplinary communication, we found that research-oriented conversation networks display more rapid growth patterns, but the conversation quality of social-oriented conversation exerts larger influence on future connections. The rapid growth of research conversation networks is predictable, as scholars from different disciplines might prioritize understanding each other’s research areas when initiating collaboration. Another possibility is that during the first two time points, scholars were in the online mode, making research topic conversations more feasible than small talk. In terms of the conversation quality, it is plausible that for each type of conversation, the corresponding first-order lagged perceived helpfulness can positively predict the network formation. For interdisciplinary teams, scholars possess different ways of thinking and different problem-solving strategies [15]. High level of perceived helpfulness implies that scholars recognize and value other fellows’ perspectives and contributions. The significantly positive effects of lagged helpfulness suggest that scholars are inclined to maintain connections they previously found helpful and valued. Interestingly, only the perceived helpfulness of small talk can influence other types of conversations beyond itself. This finding aligns with the affective primacy theory [24,25] and highlights the importance of social conversations in the building of interdisciplinary relationships. The role of interpersonal affect is valued in the collaboration process. Scholars are more likely to initiate future connections with individuals with whom they have had high-quality, non-work-related conversations. Extensive research suggests that self-disclosure plays a crucial role in the formation of close relationships [36,37]. Compared to research-oriented conversations, small talk may offer a higher degree of self-disclosure, thereby having greater power to enhance interdisciplinary communications.

### Conversation mode effects on interdisciplinary collaborations

The communication modes show different effects in three conversation networks. The conversation quality of online small talk can better foster conversations about career life and future collaborations (i.e., CCR) compared to in-person small talk. However, such moderation effect was not observed in conversations about current mHealth projects (i.e., PT) and non-research-related topics (i.e., ST). This result might be attributed to the fact that compared to in-person social conversations, online small talk provides a more structured, flexible, and private communication environment [38]. Additionally, online platforms are able to record conversation content, allowing scholars to review previous discussions and have deep information processing [27]. Such characteristics might facilitate scholars’ willingness to expand the prior informal conversations into more in-depth professional interactions in career life and future collaborations. Moreover, given that the online period represents the initial phase of relationship building among scholars (the first two time points), and that socially oriented conversations are more difficult to initiate online than in person (as evidenced by the sparsity of early ST networks), the occurrence of small talk conversations during the online phase may suggest a stronger level of interpersonal connection. This could indicate greater trust and a higher likelihood of discussing future plans and potential collaborations.

Furthermore, for research-oriented networks (i.e., PT and CCR), the perceived helpfulness of corresponding in-person conversations is more effective than that of online conversations in promoting interdisciplinary research conversations. This may be because in research-oriented conversations, it is crucial for scholars from different disciplines to ensure other fellows accurately comprehend the conveyed information. Compared to online conversations, in-person conversations enable scholars to utilize more non-verbal cues to verify understanding of listeners [39], and hence enhance the development of future research communications. In the social conversation network, it is interesting to find an interaction effect of the helpfulness of mHealth projects and training conversations. The significant interaction between PT helpfulness and communication mode may be caused by the program design, which mainly focuses on completing capstone projects within the team. Also note that after controlling the interaction terms, we can find that scholars from the same discipline are more likely to have small talk, probably because being from the same discipline can have more shared topics to initiate informal communication.

### Significance and contributions

Our study makes significant contributions to our understanding of interdisciplinary collaboration by examining the nuanced dynamics of conversations among scholars from diverse fields. The conversations among interdisciplinary scholars are unique due to the researchers’ identities and the interdisciplinary nature. The scholars’ identities make research topics one of the most important components of conversations, while the interdisciplinary nature increases the complexity of these research discussions. By employing a longitudinal network model with time-varying covariates, the study uncovers strong causal relationships and detailed functions of different conversation types in fostering interdisciplinary communication. Additionally, for interdisciplinary collaboration, cross-regional cooperation predominates. We conducted a detailed analysis of the effects of different types of conversations in both online and in-person modes, providing informative and practical guidelines for interdisciplinary communication.

A key finding in this study emphasizes the critical role of social relationships in interdisciplinary collaboration. The perceived helpfulness of social-oriented conversations emerges as a powerful factor, suggesting that knowing colleagues on a personal level significantly enhances collaborative potential. This insight underscores the importance of researchers actively participating in social events. Our research also provides valuable insights into the effects of different conversation types in both online and in-person modes, offering practical guidelines for effective interdisciplinary communication across geographical boundaries. Furthermore, the study highlights the influence of demographic factors (race, region, and age) on conversation formation, with scholars showing a tendency to interact with similar individuals. This finding emphasizes the need for intentional efforts to embrace diversity in interdisciplinary settings, ensuring a rich exchange of ideas and perspectives that can drive innovation and scientific progress. However, also notice that since the total sample size is relatively small (30 scholars), breaking down the demographic during the modeling process could introduce uncertainty in the estimates of the demographic factors.

Taking all results together, when structuring interdisciplinary training programs, it would be valuable to incorporate more social-oriented activities. Both online and in-person social activities can benefit participants in different ways, and thus both formats should be emphasized. Specifically, program designers can create more opportunities for participants to get to know each other during online training sessions. For example, they could include icebreaker activities, allocate time for extended self-introduction sections, and incorporate structured social breakout rooms during Zoom meetings to encourage informal interactions. Additionally, interactive elements such as virtual coffee breaks or group discussions about some fun non-research-related topics could help foster a sense of community (such as pair-sharing activity of what made you smile recently). For in-person training, organizers can consider holding social events after the sessions, such as dinner gatherings, or organizing tours or group activities around the training venue. These types of social events can help build trust and rapport among participants, which may facilitate future collaborations.

### Limitations and future directions

The current study is not without limitations. First, the network data collected during the online period is limited with only a single time point. The unbalanced data volume may cause the model analysis results to reflect patterns underlying the in-person mode. Second, this study did not analyze the direct impact of different types of conversations on the effectiveness of interdisciplinary collaboration outcomes. Although we provide important insights on the development of interdisciplinary relationships, it remains unclear how different types of conversations impact the team performance. This is largely because this study focuses on the early stages of relationship formation and uses this to predict future collaboration. Future research could build on this by tracking the impact of conversations on later collaboration outcomes, such as collaborative publications and jointly applied research grants. Third, only the main effects of conversation quality and interactions with communication modes were considered, while the potential interactions with time were excluded. Namely, how the role of different types of conversations evolves at different stages of the establishment of interdisciplinary relationships remains unknown. The model design without time interaction terms is because there are shifts in communication modes during the formation of interdisciplinary relationships. Simply adding a time interaction effect in the model can thus generate confounding with mode effects. Fourth, in this study, the conversation data are based on scholars’ self-reports of their interactions on the day of the study. This reliance on self-reporting introduces potential uncertainty in the accuracy of the reported conversations, as scholars may underreport or overreport their interactions due to imperfect memory. To mitigate this, we included scholars’ pictures and names in the surveys to help them recall their conversations more accurately. Despite these efforts, however, some degree of reporting error may still remain. Future researchers might consider alternative objective evidence of conversations, such as conversation recording data, to reduce recall bias. But this approach could introduce new uncertainties, such as the potential for inconsistencies in manual coding. Fifth, the conversation data in this study were collected across seven observation points within a 15-day period, representing a short-term snapshot of relationship formation. While this design was necessary due to practical constraints (e.g., the difficulty of assembling scholars over an extended period), it is important to acknowledge that the extent to which the effects of different types of conversations generalize to long-term collaboration remains an open question for future research. Lastly, caution is needed when making potential claims based on the findings. Since helpfulness ratings are only available when a conversation occurs, defining the counterfactual is challenging. Specifically, it is unclear whether the counterfactual should refer to the occurrence of a particular type of conversation or the degree of helpfulness within a conversation.

Building on findings obtained in this study, we invite further explorations into the following directions: (1) collecting detailed conversation content data through audio or video recordings during team collaboration and incorporating qualitative analyses or advanced natural language processing techniques to provide a richer understanding of the conversation dynamics. Such analyses can help reveal the factors that influence the perceived helpfulness of conversations. For example, during the conversations, examining the frequency of keywords related to future collaboration, analyzing how often participants take turns expressing their viewpoints, and exploring how they help other scholars understand their research fields; (2) analyzing the underlying mechanism of the small talk effect. Based on the current findings, small talk brings robust improvements in all types of conversations. It is fruitful to investigate what aspects of small talk are most valuable, such as will topics sharing positive experiences have similar effects with topics discussing negative experiences; (3) examining the impact of different forms of online communication on interdisciplinary team collaboration. This study focuses on the virtual interactions between scholars, but for distributed interdisciplinary teams, there are various forms of online communication, such as email and texts. Understanding different effects of communication forms on team formation and functioning could be an interesting avenue; (4) applying and validating the research findings beyond the mHealth training program are valuable. Although this study was conducted within mHealth training institutes, the analytical framework and results have the potential to be applied to different training programs (e.g., the Science Dialog Program [40]) that capture participant conversations. Validating these findings across different fields with interdisciplinary collaboration as an important feature would be meaningful, such as education, public health, or environmental science. These fields could also benefit from exploring how communication patterns among participants influence collaborative outcomes. Additionally, although the interaction between CCR helpfulness and small talk helpfulness showed a significant impact (*p* < 0.01) on the development of CCR conversations, it remains unclear whether this conclusion can be generalized to fields outside of mHealth. Validating this conclusion in different settings would be meaningful; (5) incorporating longitudinal follow-ups to assess how early-stage interactions may influence sustained collaboration, including the formation of joint publications, grants, or other scholarly outputs.

## Supporting information

10.1017/cts.2025.10124.sm001 Huang et al. supplementary material Huang et al. supplementary material

## Data Availability

The analytic codes of this study are available on: https://osf.io/37wbj/?view_only=2fe6d23decb24dbf8fa34fac486c4525. The data are not publicly available due to privacy and ethical restrictions.

## Supplementary material

The supplementary material for this article can be found at https://doi.org/10.1017/cts.2025.10124
