# Supplementary material for: The power of social talk: A longitudinal network analysis of conversations in fostering interdisciplinary collaboration
Source: J Clin Transl Sci. 2025 Aug 13;9(1):e194. doi: 10.1017/cts.2025.10124 (PMC12444692; doi:10.1017/cts.2025.10124)

**Supplementary materials: The power of social talk: A longitudinal network analysis of conversations in fostering interdisciplinary collaboration**

Contents

[Table S1. Model parameter estimates and standard errors (SE) with balanced-waves subsamples (i.e., 2 online waves and 2 in-person waves) for Project and Training (PT), Career, Collaboration, and Research (CCR), and Small Talk (ST) conversation networks. 1](#_Toc199619302)

[Table S2. Model 1 parameter estimates and standard errors (SE) with differential homophily for Project and Training (PT), Career, Collaboration, and Research (CCR), and Small Talk (ST) conversation networks. 2](#_Toc199619303)

[Table S3. Model 2 parameter estimates and standard errors (SE) with differential homophily for Project and Training (PT), Career, Collaboration, and Research (CCR), and Small Talk (ST) conversation networks. 4](#_Toc199619304)

[Figure S1. Project and Training (PT) conversation network (online mode: June 5 and June 26; in-person mode: July 16 to July 20). 6](#_Toc199619305)

[Figure S2. Career, Collaboration, and Research (CCR) conversation network (online mode: June 5 and June 26; in-person mode: July 16 to July 20). 7](#_Toc199619306)

[Figure S3. Small Talk (ST) conversation network (online mode: June 5 and June 26; in-person mode: July 16 to July 20). 8](#_Toc199619307)

Table S1. Model parameter estimates and standard errors (SE) with balanced-waves subsamples (i.e., 2 online waves and 2 in-person waves) for Project and Training (PT), Career, Collaboration, and Research (CCR), and Small Talk (ST) conversation networks.

| **Model 2** | PT | | CCR | | ST | |
| --- | --- | --- | --- | --- | --- | --- |
| Effect | estimates | SE | estimates | SE | estimates | SE |
| edges | **-4.767***** | 0.194 | **-4.425***** | 0.160 | **-4.077***** | 0.154 |
| mutual | **1.833***** | 0.347 | **0.860**** | 0.313 | **0.770**** | 0.250 |
| cyclicalties | **-0.788***** | 0.157 | **-0.356**** | 0.118 | **-0.224*** | 0.090 |
| transitiveties | **1.520***** | 0.151 | **1.402***** | 0.129 | **1.246***** | 0.124 |
| team | **2.573***** | 0.225 | **1.920***** | 0.170 | **1.764***** | 0.156 |
| region | 0.318 | 0.192 | 0.261 | 0.175 | 0.086 | 0.156 |
| race | **0.448**** | 0.169 | 0.273 | 0.158 | 0.232 | 0.129 |
| gender | 0.207 | 0.145 | 0.174 | 0.133 | 0.049 | 0.116 |
| age | 0.243 | 0.155 | 0.253 | 0.151 | **0.298*** | 0.123 |
| discipline | -0.248 | 0.196 | 0.043 | 0.184 | 0.197 | 0.143 |
| PT helpfulness | -0.030 | 0.078 | **-0.184*** | 0.088 | -0.130 | 0.077 |
| CCR helpfulness | 0.020 | 0.132 | 0.039 | 0.141 | -0.044 | 0.141 |
| ST helpfulness | **0.320*** | 0.149 | **0.544***** | 0.146 | **0.592***** | 0.167 |
| PT helpfulness*mode | **0.352**** | 0.124 | **0.290**** | 0.111 | **0.271**** | 0.104 |
| CCR helpfulness*mode | -0.156 | 0.172 | 0.313 | 0.168 | -0.073 | 0.169 |
| ST helpfulness*mode | -0.059 | 0.179 | **-0.569***** | 0.167 | -0.193 | 0.183 |

*Note*. *** indicates *p*<.001, ** indicates *p*<.01, * indicates *p*<.05. The significant estimates are highlighted. (online mode = 0, in-person mode = 1)

Table S2. Model 1 parameter estimates and standard errors (SE) with differential homophily for Project and Training (PT), Career, Collaboration, and Research (CCR), and Small Talk (ST) conversation networks.

| **Model 1** | PT | | CCR | | ST | |
| --- | --- | --- | --- | --- | --- | --- |
| Effect | estimates | SE | estimates | SE | estimates | SE |
| edges | **-4.813***** | 0.171 | **-4.440***** | 0.142 | **-3.917***** | 0.128 |
| mutual | **1.882***** | 0.285 | **1.092***** | 0.240 | **1.083***** | 0.190 |
| cyclicalties | **-0.917***** | 0.131 | **-0.404***** | 0.096 | **-0.322***** | 0.073 |
| transitiveties | **1.428***** | 0.126 | **1.277***** | 0.105 | **1.022***** | 0.095 |
| **team** |  |  |  |  |  |  |
| Team 1 | **2.925***** | 0.282 | **1.945***** | 0.231 | **1.699***** | 0.218 |
| Team 2 | **2.837***** | 0.282 | **2.115***** | 0.228 | **1.624***** | 0.205 |
| Team 3 | **2.888***** | 0.265 | **2.069***** | 0.216 | **1.910***** | 0.215 |
| Team 4 | **2.710***** | 0.279 | **1.690***** | 0.207 | **1.533***** | 0.204 |
| Team 5 | **3.854***** | 0.414 | **2.244***** | 0.265 | **2.226***** | 0.281 |
| Team 6 | **2.924***** | 0.308 | **1.869***** | 0.267 | **2.160***** | 0.269 |
| **region** |  |  |  |  |  |  |
| Northeast | -0.863 | 0.515 | -0.303 | 0.396 | -0.472 | 0.337 |
| Southeast | **0.621**** | 0.189 | **0.513**** | 0.163 | **0.427**** | 0.138 |
| Southwest | **2.118***** | 0.402 | **2.101***** | 0.335 | **1.665***** | 0.345 |
| Midwest | - | - | - | - | - | - |
| West | -0.147 | 0.726 | -0.433 | 0.753 | -0.983 | 0.736 |
| International | **-2.501*** | 0.975 | **-1.982*** | 0.887 | **-1.733*** | 0.823 |
| Missing | - | - | - | - | - | - |
| **race** |  |  |  |  |  |  |
| Black/African American | -0.316 | 0.547 | 0.411 | 0.461 | 0.449 | 0.436 |
| White/Caucasian | **0.391*** | 0.190 | -0.020 | 0.177 | 0.207 | 0.133 |
| Hispanic/Latina/Latino | **1.079*** | 0.472 | **1.445***** | 0.316 | **0.863*** | 0.344 |
| Asian | **0.852***** | 0.219 | **0.683***** | 0.196 | **0.488**** | 0.175 |
| Prefer not to state | - | - | - | - | - | - |
| Missing | - | - | - | - | - | - |
| **gender** |  |  |  |  |  |  |
| Male | 0.159 | 0.221 | 0.068 | 0.197 | 0.074 | 0.169 |
| Female | 0.117 | 0.137 | 0.030 | 0.124 | 0.015 | 0.103 |
| Prefer not to state | - | - | - | - | - | - |
| Missing | - | - | - | - | - | - |
| **age** |  |  |  |  |  |  |
| 29 or under | - | - | - | - | - | - |
| 30 to 39 | 0.141 | 0.159 | 0.229 | 0.141 | **0.371**** | 0.118 |
| 40 to 49 | 0.417 | 0.214 | 0.346 | 0.192 | 0.190 | 0.167 |
| Missing | - | - | - | - | - | - |
| **discipline** |  |  |  |  |  |  |
| Psychology | -0.202 | 0.207 | 0.060 | 0.178 | 0.074 | 0.145 |
| Medicine / Nursing | 0.551 | 0.449 | **0.826*** | 0.366 | 0.478 | 0.341 |
| CS / Engineering / Data Science | **0.854**** | 0.323 | 0.560 | 0.321 | 0.449 | 0.281 |
| Public Health / Others | 0.039 | 0.605 | 0.260 | 0.520 | **0.974**** | 0.300 |
| Missing | - | - | - | - | - | - |
| PT helpfulness | **0.148**** | 0.054 | -0.058 | 0.050 | 0.019 | 0.051 |
| CCR helpfulness | -0.010 | 0.059 | **0.303***** | 0.052 | -0.047 | 0.053 |
| ST helpfulness | **0.298***** | 0.057 | **0.175***** | 0.049 | **0.463***** | 0.047 |

*Note*. *** indicates *p*<.001, ** indicates *p*<.01, * indicates *p*<.05. The significant estimates are highlighted. “-” denotes missing estimates due to insufficient observations.

Table S3. Model 2 parameter estimates and standard errors (SE) with differential homophily for Project and Training (PT), Career, Collaboration, and Research (CCR), and Small Talk (ST) conversation networks.

| **Model 2** | PT | | CCR | | ST | |
| --- | --- | --- | --- | --- | --- | --- |
| Effect | estimates | SE | estimates | SE | estimates | SE |
| edges | **-4.819***** | 0.172 | **-4.419***** | 0.143 | **-3.909***** | 0.129 |
| mutual | **1.694***** | 0.288 | **1.053***** | 0.247 | **1.029***** | 0.196 |
| cyclicalties | **-0.859***** | 0.128 | **-0.423***** | 0.098 | **-0.324***** | 0.073 |
| transitiveties | **1.433***** | 0.127 | **1.269***** | 0.109 | **1.022***** | 0.099 |
| **team** |  |  |  |  |  |  |
| Team 1 | **3.036***** | 0.284 | **2.104***** | 0.228 | **1.840***** | 0.221 |
| Team 2 | **2.897***** | 0.281 | **2.168***** | 0.226 | **1.679***** | 0.207 |
| Team 3 | **2.854***** | 0.258 | **2.111***** | 0.225 | **1.895***** | 0.216 |
| Team 4 | **2.778***** | 0.272 | **1.735***** | 0.215 | **1.574***** | 0.216 |
| Team 5 | **4.420***** | 0.433 | **2.434***** | 0.284 | **2.559***** | 0.307 |
| Team 6 | **3.174***** | 0.317 | **2.061***** | 0.274 | **2.332***** | 0.286 |
| **region** |  |  |  |  |  |  |
| Northeast | -0.867 | 0.535 | -0.305 | 0.404 | -0.479 | 0.357 |
| Southeast | **0.616**** | 0.192 | **0.534**** | 0.166 | **0.436**** | 0.135 |
| Southwest | **2.163***** | 0.406 | **2.137***** | 0.334 | **1.706***** | 0.339 |
| Midwest | - | - | - | - | - | - |
| West | -0.157 | 0.760 | -0.460 | 0.743 | -0.998 | 0.745 |
| International | **-2.677**** | 0.981 | **-2.221*** | 0.898 | **-1.873*** | 0.823 |
| Missing | - | - | - | - | - | - |
| **race** |  |  |  |  |  |  |
| Black/African American | -0.188 | 0.562 | 0.495 | 0.441 | 0.559 | 0.448 |
| White/Caucasian | **0.400*** | 0.198 | -0.018 | 0.182 | 0.215 | 0.136 |
| Hispanic/Latina/Latino | **1.155*** | 0.490 | **1.460***** | 0.321 | **0.913**** | 0.350 |
| Asian | **0.854***** | 0.222 | **0.717***** | 0.192 | **0.510**** | 0.184 |
| Prefer not to state | - | - | - | - | - | - |
| Missing | - | - | - | - | - | - |
| **gender** |  |  |  |  |  |  |
| Male | 0.207 | 0.225 | 0.076 | 0.201 | 0.090 | 0.168 |
| Female | 0.080 | 0.140 | 0.010 | 0.125 | 0.003 | 0.104 |
| Prefer not to state | - | - | - | - | - | - |
| Missing | - | - | - | - | - | - |
| **age** |  |  |  |  |  |  |
| 29 or under | - | - | - | - | - | - |
| 30 to 39 | 0.172 | 0.160 | 0.238 | 0.144 | 0.396 | 0.121 |
| 40 to 49 | **0.448*** | 0.220 | 0.363 | 0.194 | **0.191**** | 0.165 |
| Missing | - | - | - | - | - | - |
| **discipline** |  |  |  |  |  |  |
| Psychology | -0.126 | 0.211 | 0.110 | 0.183 | 0.100 | 0.147 |
| Medicine / Nursing | 0.514 | 0.458 | **0.821*** | 0.361 | 0.471 | 0.351 |
| CS / Engineering / Data Science | **0.871**** | 0.328 | 0.544 | 0.322 | 0.445 | 0.284 |
| Public Health / Others | 0.098 | 0.615 | 0.263 | 0.522 | **1.004***** | 0.303 |
| Missing | - | - | - | - | - | - |
| PT helpfulness | -0.164 | 0.089 | **-0.205*** | 0.094 | **-0.220*** | 0.094 |
| CCR helpfulness | 0.110 | 0.144 | 0.045 | 0.167 | 0.011 | 0.151 |
| ST helpfulness | 0.303^a^ | 0.155 | **0.554**** | 0.173 | **0.590***** | 0.161 |
| PT helpfulness*mode | **0.506***** | 0.107 | **0.228*** | 0.103 | **0.335**** | 0.102 |
| CCR helpfulness*mode | -0.160 | 0.161 | 0.290 | 0.178 | -0.089 | 0.162 |
| ST helpfulness*mode | -0.125 | 0.169 | **-0.485**** | 0.183 | -0.181 | 0.168 |

*Note*. *** indicates *p*<.001, ** indicates *p*<.01, * indicates *p*<.05. The significant estimates are highlighted. a: *p*=0.0504. “-” denotes missing estimates due to insufficient observations. (online mode = 0, in-person mode = 1)

Figure S1. Project and Training (PT) conversation network (online mode: June 5 and June 26; in-person mode: July 16 to July 20).


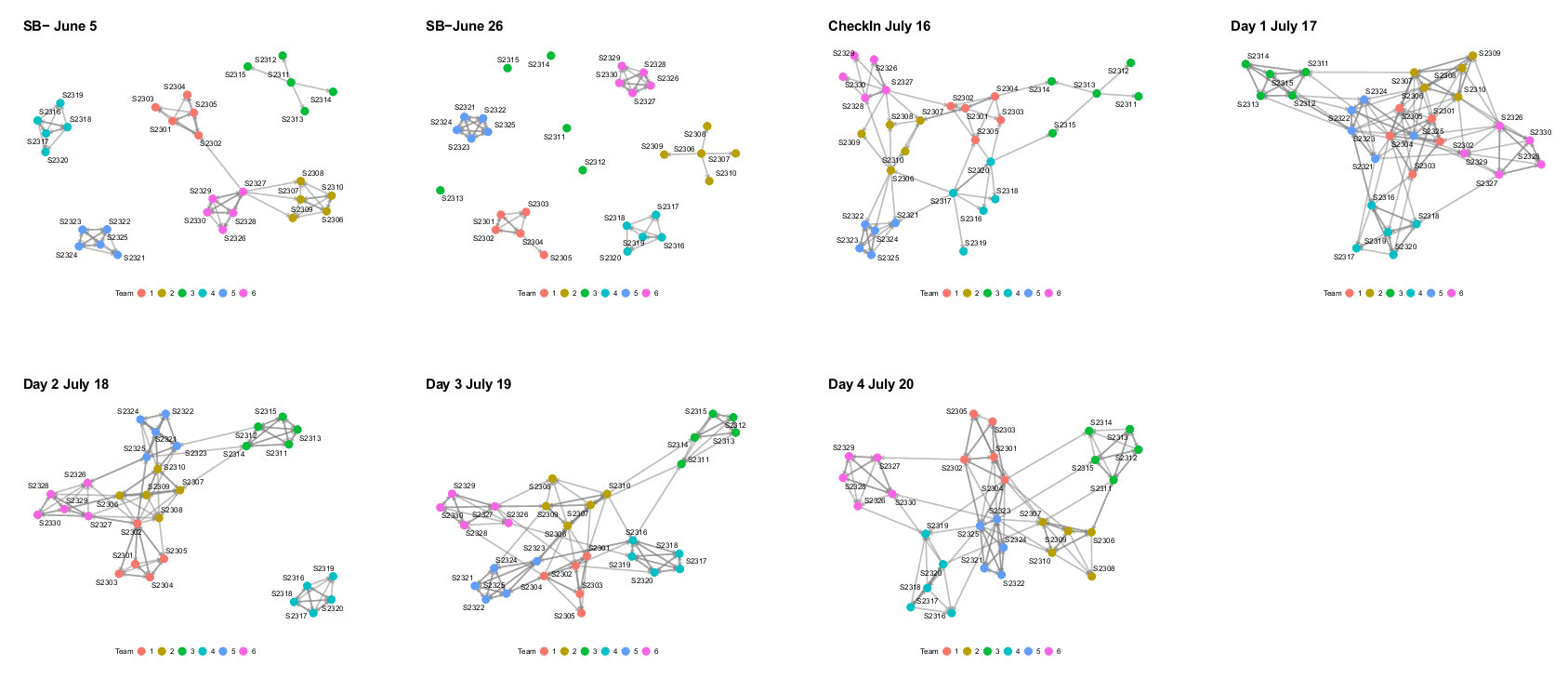


Figure S2. Career, Collaboration, and Research (CCR) conversation network (online mode: June 5 and June 26; in-person mode: July 16 to July 20).


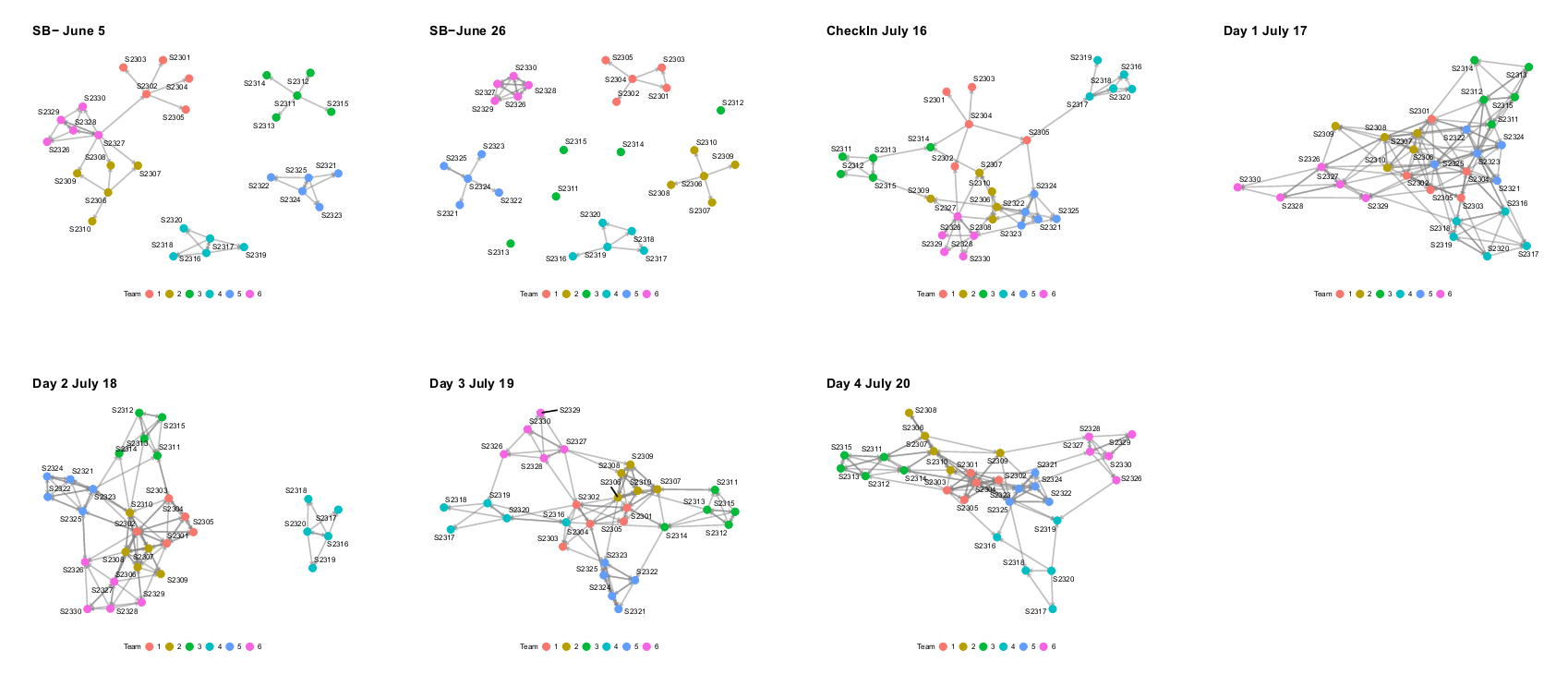


Figure S3. Small Talk (ST) conversation network (online mode: June 5 and June 26; in-person mode: July 16 to July 20).
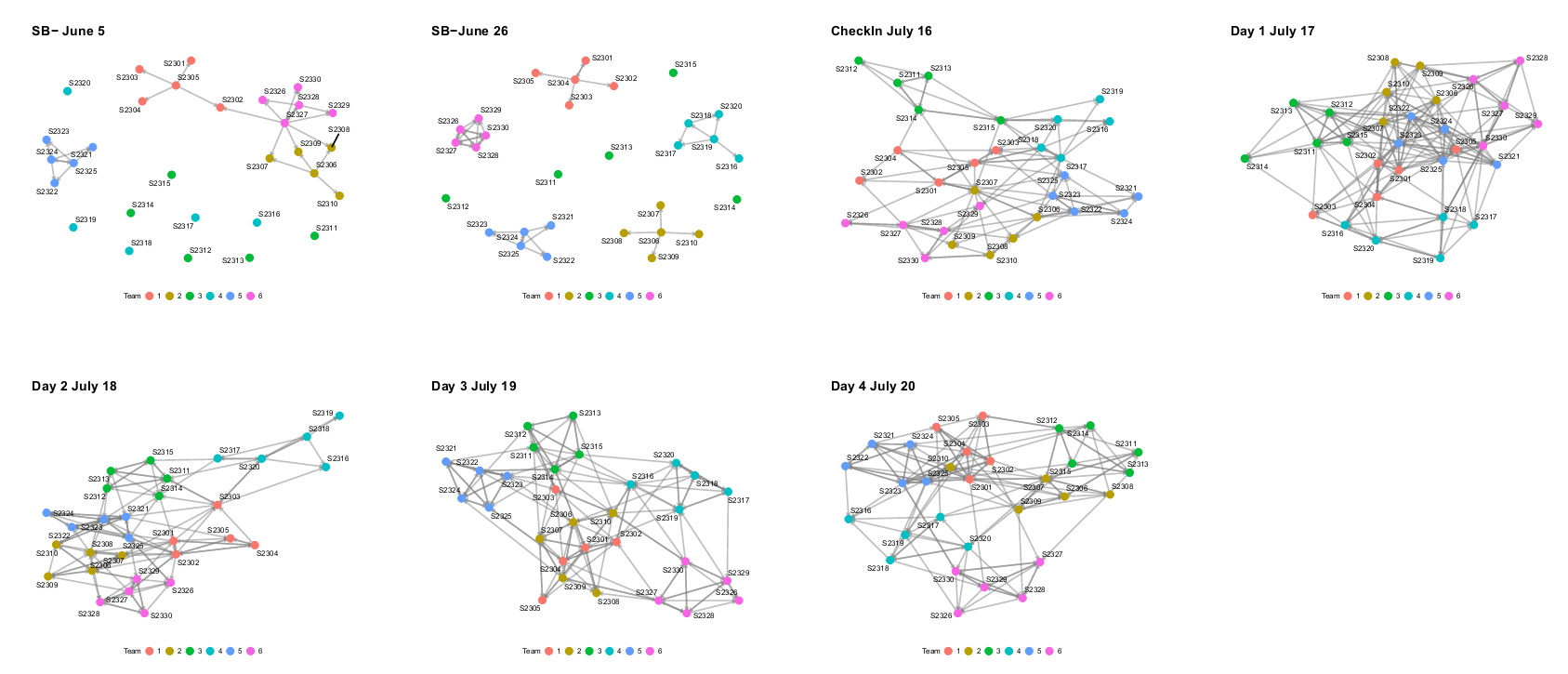

Supplement: Huang et al. supplementary material [file S2059866125101246sup001.docx]
